# Endometrial thickness/volume and uterine peristalsis in adenomyosis patients undergoing frozen embryo transfer to predict pregnancy outcomes: A retrospective study

**DOI:** 10.1097/MD.0000000000044907

**Published:** 2025-09-26

**Authors:** Xue Ke, Xuefei Liang, Meixian Wang, Xiaoguang Shao

**Affiliations:** a Graduate School of Dalian Medical University, Dalian Medical University, Dalian, Liaoning, China; b Department of Reproduction and Infertility, Chengdu Women’s and Children’s Central Hospital, School of Medicine, University of Electronic Science and Technology of China, Chengdu, China; c Reproductive Center, Affiliated Zhongshan Hospital of Dalian University, Dalian, Liaoning, China.

**Keywords:** adenomyosis, endometrial receptivity, endometrial thickness, endometrial volume, pregnancy outcome, uterine peristalsis

## Abstract

To analyze the significance of endometrial thickness (EMT), endometrial volume and uterine peristalsis (UP) for forecasting the pregnancy outcome of patients with adenomyosis (ADM) undergoing frozen embryo transfer (FET). Women diagnosed with ADM between February 15, 2017, and March 15, 2025 from one tertiary care center in southwest China, were retrospectively included. There were 111 cases in the pregnant group, whereas 60 in the nonpregnant group. EMT, endometrial volume and UP on the endometrial transformation day and 1 day before embryo transfer (ET) during the FET cycle between 2 groups were compared. No significant differences could be observed in EMT or endometrial volume on the endometrial transformation day or 1 day before ET in 2 groups (*P* = .288, *P* = .461, *P* = .913, *P* = .239, separately). The nonpregnant group had an increased UP frequency relative to the pregnant group on the endometrial transformation day (2.28 ± 1.32 vs 1.29 ± 0.95, *P* = .001) and 1 day before ET (1.82 ± 0.97 vs 1.36 ± 0.94, *P* = .003). Binary logistic regression analysis revealed that EMT and endometrial volume at 2 time points were not the independent risk factors affecting the FET outcome (odds ratios: 1.112, 95% confidence interval [CI]: 0.888–1.393, *P* = .355; 1.140, 95% CI: 0.834–1.558, *P* = .411; 0.902, 95% CI: 0.717–1.136, *P* = .381; 1.201, 95% CI: 0.937–1.538, *P* = .149). As suggested by receiver operating characteristic curve analysis, the area under the curve values of EMT and endometrial volume on the endometrial transformation day were 0.461 and 0.458, while those 1 day before ET were 0.501 and 0.444 separately. Nonetheless, UP on the endometrial transformation day was identified to be a factor independently affecting the clinical pregnancy probability of ADM patients, and the odds ratio was 0.323 (95% CI:0.187–0.557). Area under the curve values of UP were 0.720 and 0.627 on the endometrial transformation day and 1 day before ET separately. Neither EMT nor endometrial volume can predict the pregnancy outcome of ADM patients undergoing FET. Uterine peristalsis in the transplantation process serves as an indicator for pregnancy outcome.

## 1. Introduction

Adenomyosis (ADM) presents with abnormal deep locations of endometrial glands and stroma in myometrium, leading to dysmenorrhea, increased menstrual volume, and infertility. ADM is a common cause of assisted pregnancy through in vitro fertilization (IVF).^[1,2]^ The IVF embryo implantation rate in patients with ADM is significantly lower than that in patients with non-uterine factor infertility, and the risk of early pregnancy loss also increases.^[3]^

Embryo quality and good endometrial receptivity are the key factors determining embryo transplantation. As suggested by the updated DNA evidence, ADM is derived from the invagination of endometrial basal layer in myometrium.^[4,5]^ In the case of ADM, the changed hormonal microenvironment can promote endometrial tissue invasion in myometrium.^[6]^ Therefore, we proposed to investigate if endometrial invasion influenced endometrial thickness (EMT) and volume among ADM patients. Some authors suggest that three-dimensional (3D) vaginal ultrasound can accurately predict endometrial receptivity. Ultrasound parameters for endometrial receptivity, like EMT, endometrial pattern, endometrial volume, Doppler ultrasound of uterine arteries, endometrial blood flow, and uterine peristalsis (UP), are identified as the predictors of pregnancy and live birth.^[7,8]^

EMT is a common index used to evaluate endometrial receptivity. Currently, consensus on whether EMT is able to forecast pregnancy outcome of assisted reproductive technology (ART) has not been reached, it is suggested in many studies that the thin endometrium is related to the low pregnancy rate.^[9,10]^ The thickness ≥ 7 mm has been demonstrated in some research to be related to an increased successful pregnancy rate, however, other studies discover that the thickness ≥ 9 mm benefits appropriate implantation and the final successful ART.^[11]^

Endometrial volume is an overlooked ultrasonic indicator for endometrial receptivity, which can comprehensively represent the whole endometrium compared with EMT at a specific part. The 3D ultrasound measurement of endometrial volume may be used to replace EMT, since it analyzes the entire endometrium rather than one certain plane. Recently, as ultrasound develops, an increasing number of studies are conducted to investigate the relation of endometrial volume with embryo implantation.^[12]^ Currently, only few studies focus on the relationships of EMT and endometrial volume in ADM patients with their pregnancy outcomes.

The nonpregnant uterus is characterized by inherent contractility, which aggressively participates in early reproduction. Uterine contractility displays the typical features of junctional zone (JZ)-originating endometrial waves and great variability in menstrual cycle. After endometrial glands and stroma invaginate at a JZ level, the neighboring myometrial tissues may experience hyperplasia, thereby generating the abnormal ultrastructure with calcium channel dysfunction that can change UP.^[13]^ The present work assessed the significance of trans-vaginal 3D Doppler ultrasound indices of EMT, endometrial volume and UP for forecasting the pregnancy outcomes in ADM patients undergoing frozen embryo transfer (FET).

## 2. Materials and methods

### 2.1. Study design and patients

This retrospective cohort study was conducted on infertile women with ADM at a tertiary care academic medical center in southwestern China. All cycles classified as ADM by pelvic plain scan magnetic resonance imaging or endometriosis related ultrasound from February 15, 2017, to March 15, 2025, were enrolled.

Our patients included were 25 to 42 years old, developed primary or secondary infertility, and were candidates of FET cycles. ADM was their main diagnosis, without other factors like male factors and ovarian reserve dysfunction. Patients below were excluded: those with a repeated abortion history, those with uterine adhesions or malformations, and endocrinological abnormalities, and those with recurrent implantation failure (>3 times).

This work gained approval from the Scientific Ethics Committee of Chengdu Women’s and Children’s Central Hospital (approval number: 2023-132) and was performed following the Declaration of Helsinki. Patients undergoing endometrial receptivity ultrasound testing provided written informed consent.

### 2.2. ADM diagnosis and classification

Morphological uterus sonographic assessment is the standard approach to recognize representative ADM characteristics during ultrasound examinations. The characteristics include asymmetrical uterine wall thickening, intramyometrial cysts or/and hyperechoic islands, fan-shaped shadowing on myometrium, myometrial echogenic sub-endometrial lines/buds, trans-lesional vascularity, and the irregular or interrupted JZ. The characteristics are modified by the same group, with those including cysts, hyperechogenic islands and/or echogenic sub-endometrial line bubs being deemed to be diagnostic characteristics, whereas others being suspicious characteristics for ADM.^[14]^

The criteria for ADM were assessed based on magnetic resonance imaging.^[15–18]^: I, intrinsic (ADM affecting the inner uterine layer but not the outer layer); II, extrinsic (ADM affecting the outer uterine layer but not its inner layer); III, intramural (ADM located solitarily with no influence on the JZ and serosa); and IV, indeterminate (ADM not corresponding to subtypes I–III).

### 2.3. Measurement procedures of EMT, endometrial volume, and UP

**Figure 1.**
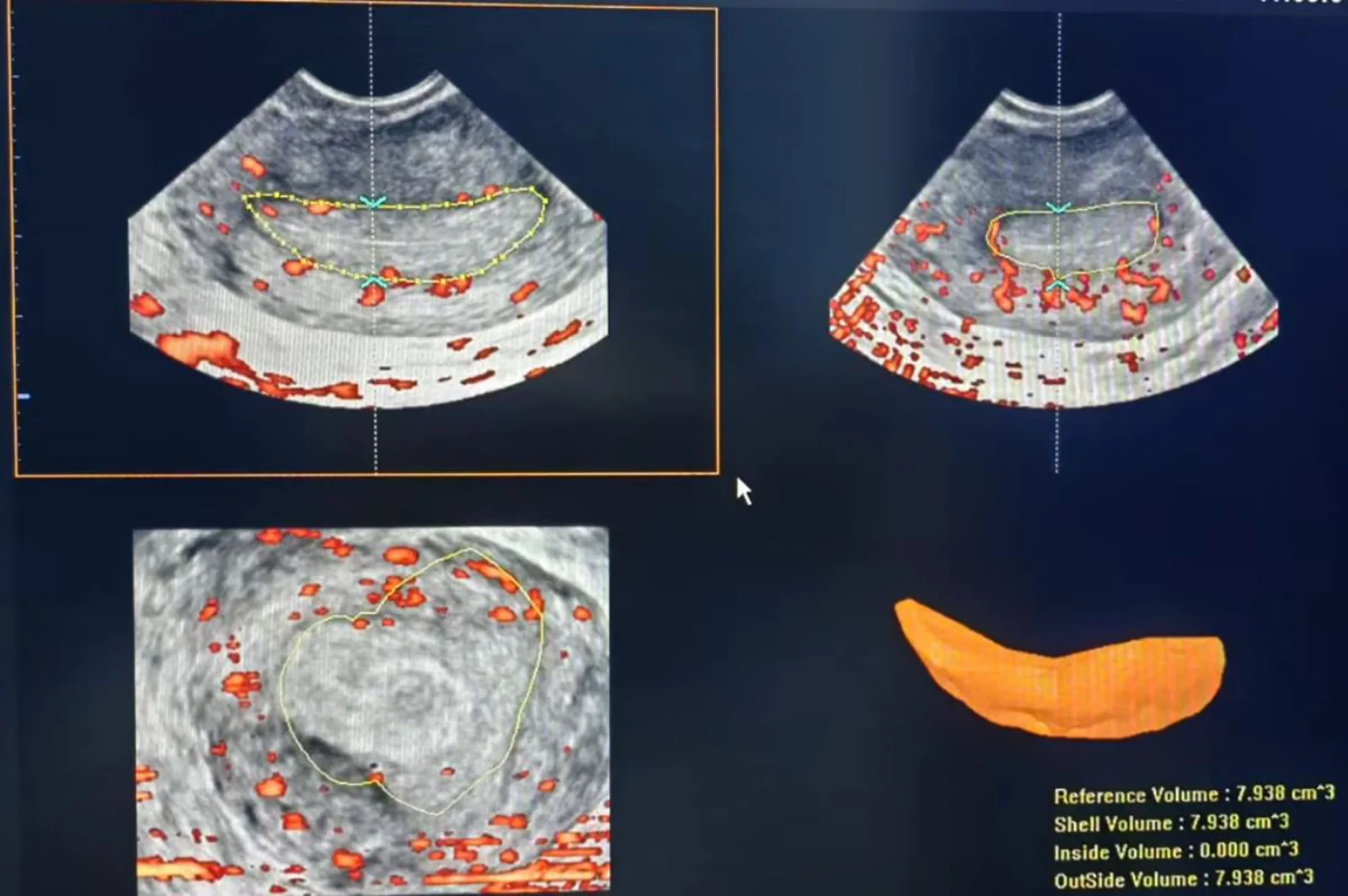
Measurement of endometrial volume in patients with ADM using 3D ultrasound. 3D = three-dimensional, ADM = adenomyosis.

A H60 3D ultrasound examination instrument (produced in South Korea, with the vaginal probe frequency of 4–9 MHz) was utilized in vaginal ultrasound examination. The uterine position was observed, and the EMT was determined in the longitudinal uterine section. The scanning area and range were adjusted under the stereoscopic reconstruction function, 3D images were acquired, and the volume measurement function button was activated. The angle between each layer during the extraction process was set to 30 degrees. After determining the image reference point, the trajectory ball was used to outline each layer. The outlining range of the uterine volume included the junction between the myometrium (between endometrial fundus and internal cervical os) and the endometrium. After the outlining was completed, the execution function button was activated, and the 3D ultrasound instrument would automatically calculate and display the results (Fig. 1). Uterine peristalsis was recorded for a 4-minute duration on the mid-sagittal plane.^[19,20]^

### 2.4. Outcome measures

Our primary outcome included clinical pregnancy rate. A biochemical pregnancy was confirmed based on a positive human chorionic gonadotropic beta result 14 days following embryo transfer (ET), but without gestational sac after 2 weeks. The clinical pregnancy referred to the intrauterine gestational sac existing 4 weeks after ET.

### 2.5. Statistical analysis

Statistical analysis was completed with SPSS 26.0 software (IBM Corp., Armonk). Meanwhile, data normality was tested by Shapiro–Wilk (S–W) test. Considering the abnormal distribution, measurement data were represented by median (25th percentile, 75th percentile) M (P25, P75), and between-group differences were analyzed by Kruscarl–Wallis H (K) test. Count data were represented by composition ratio, and compared by Pearson Chi-square test. Factors independently affecting clinical pregnancy were determined by binary logistic regression after confounders were adjusted. Moreover, receiver operating characteristic curve and area under the curve (AUC) analyses were carried out for determining the prediction performance. Notably, the AUC values of 1, >0.9, and 0.7 to 0.9 indicate the perfect test, high accuracy, and moderate accuracy separately. Besides, 95% confidence intervals (CIs) and *P*-values were determined, with *P* < .05 suggesting significant differences.

## 3. Results

From February 15, 2017 to March 15, 2025, a total of 181 ADM patients at the reproductive center of our hospital underwent the FET cycles. Among them, 4 cases with uterine malformation, 4 receiving surgery for endometrial adhesions and 2 with a history of recurrent miscarriage were excluded. Finally, 171 patients were enrolled, including 111 in the pregnant group, and 60 in the nonpregnant group. Various variables were assessed in these patients, including age, body mass index, infertility duration, cause of infertility, anti-Mullerian hormone (AMH), and follicle-promoting hormone (FSH) contents, and the embryos transferred number.

### 3.1. Differences in demographic characteristics between the 2 groups

From Table 1, age, body mass index, infertility duration, type, and cause of infertility were not significantly different between the 2 groups. Meanwhile, basal hormonal contents, like basal estradiol (E2), FSH, and luteinizing hormone were not significantly different. But in terms of the ovarian reserve, AMH and antral follicular count levels were significantly different between the 2 groups (3.14 ± 2.49 ng/mL vs 2.08 ± 1.03, *P* = .046; 11.80 ± 6.17 vs 9.43 ± 5.98, *P* = .017). But the embryos transferred number and ET type showed no significant difference in 2 groups.

**Table 1:** Baseline and cycle characteristics of the 2 groups.

Characteristic	Pregnant group	Nonpregnant group	*P*-value
Number of patients	111	60	
Age (yr)	33.27 ± 3.98	34.52 ± 4.63	.67
BMI (kg/m^2^)	22.60 ± 2.78	22.65 ± 2.78	.915
*Type of infertility*			.257
1ry	41 (36.9)	17 (28.3)	
2ry	70 (63.1)	43 (71.7)	
AMH (ng/mL)	3.14 ± 2.49	2.08 ± 1.03	.046
Basal FSH (mIU/mL)	6.87 ± 3.05	7.70 ± 3.25	.097
Basal LH (mIU/mL)	3.44 ± 2.56	2.82 ± 2.36	.125
Basal E2 (pg/mL)	40.56 ± 23.69	42.24 ± 31.19	.693
Basal P (ng/mL)	0.43 ± 0.18	0.47 ± 0.25	.280
CA125 (U/L)	29.33 (16.73–41.13)	25.30(13.60–39.80)	.193
AFC	11.80 ± 6.17	9.43 ± 5.98	.017
Duration of infertility (yr)	4.06 ± 3.38	4.85 ± 3.99	.172
Uterine anteroposterior diameter (cm)	4.80 ± 1.34	5.11 ± 1.39	.149
*Embryos transferred*			.090
Cleavage stage embryo	67 (60.4)	44 (73.3)	
Blastocyst	44 (39.6)	16 (26.7)	
Number of embryos transferred	1.66 ± 0.56	1.57 ± 0.50	.266

Values are presented as mean ± standard deviation or number (%).

AFC = antral follicular count, AMH = anti-Müllerian hormone, BMI = body mass index, DOR = diminished ovarian reserve, E2 = estradiol, FSH = follicle-stimulating hormone, LH = luteinizing hormone.

### 3.2. Comparisons of EMT, endometrial volume, and UP between 2 groups

On the endometrial transformation day and 1 day before ET, 3D ultrasound examinations were performed on ADM patients (Table 2). EMT and endometrial volume were not significantly different between 2 groups on the endometrial transformation day or 1 day before ET (*P* = .288, *P* = .461, *P* = .913, *P* = .239, separately). The nonpregnant group had an increased UP frequency relative to the pregnant group on the endometrial transformation day (2.28 ± 1.32 vs 1.29 ± 0.95, *P* = .001) and 1 day before ET(1.82 ± 0.97 vs 1.36 ± 0.94, *P* = .003).

**Table 2:** Comparing endometrial thickness, endometrial volume, and uterine peristalsis of 2 groups on the endometrial transformation day and 1 day prior to FET.

	Day of endometrial transformation		*P*-value	1 day prior to FET		*P*-value
Characteristic	Pregnant group	Nonpregnant group		Pregnant group	Nonpregnant group	
Number of patients	111	60		111	60	
EMT (mm)	9.93 ± 2.17	9.58 ± 1.81	.288	9.46 ± 1.84	9.42 ± 1.55	.913
Endometrial volume (cm^3^)	3.50 ± 1.35	3.34 ± 1.48	.461	3.52 ± 1.76	3.20 ± 1.55	.239
Uterine peristalsis	1.29 ± 0.95	2.28 ± 1.32	.001	1.36 ± 0.94	1.82 ± 0.97	.003

Values are presented as mean ± standard deviation or number (%).

EMT = endometrial thickness, FET = frozen embryo transfer.

### 3.3. Analysis of parameters affecting clinical pregnancy in patients with ADM

**Figure 2.**
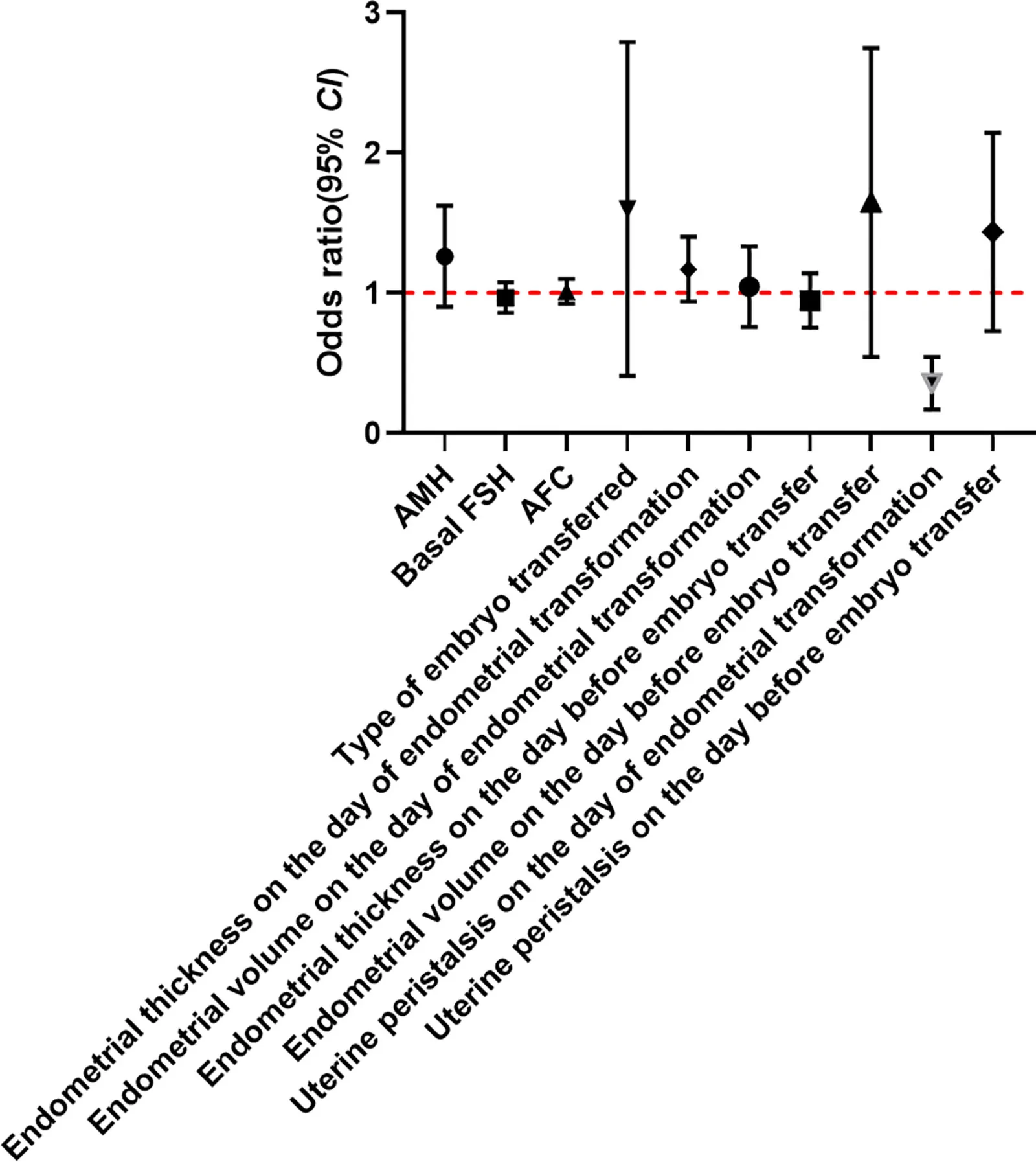
Binary stepwise logistic regression analysis of covariates on the pregnancy outcomes of ADM patients. ADM = adenomyosis.

Binary stepwise logistic regression analysis was conducted for determining whether EMT, endometrial volume, and UP independently influenced the pregnancy outcomes of ADM patients. When the AMH, antral follicular count, Basal FSH, type of embryos transferred, EMT, and endometrial volume were included as covariates, these variables were not significantly associated with pregnancy outcomes (Fig. 2). EMT on the endometrial transformation day and 1 day before ET was not the significant factor for pregnancy outcome of ADM patients (odds ratios [ORs]: 1.112, 95% CI: 0.888–1.393, *P* = .355; 0.902, 95% CI: 0.717–1.136, *P* = .381). Moreover, endometrial volume on the endometrial transformation day and 1 day before ET was not the significant factor for pregnancy outcome of ADM patients (ORs: 1.140, 95% CI: 0.834–1.558, *P* = 0. 411; 1.201, 95% CI: 0.937–1.538, *P* = .149). Uterine peristalsis on the endometrial transformation day, rather than that 1 day before ET (95% CI: 0.187–0.557, *P* = 0. 282), was the factor independently affecting clinical pregnancy in ADM patients, and the OR was 0.323 (95% CI:0.187–0.557).

**Figure 3.**
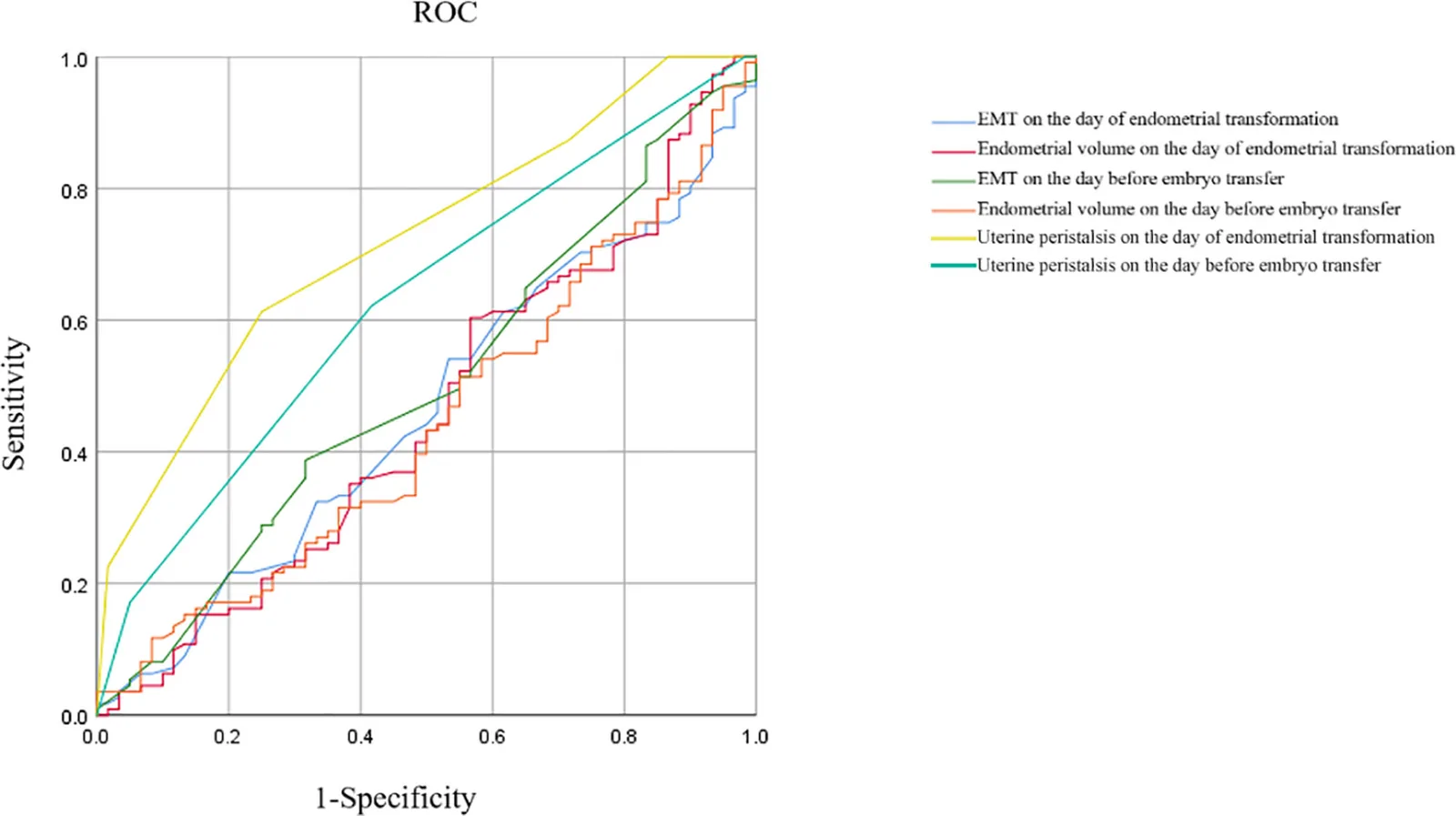
ROC curves of EMT, endometrial volume and uterine peristalsis on the endometrial transformation day and 1 day prior to ET in ADM patients. ADM = adenomyosis, EMT = endometrial thickness, ROC = receiver operating characteristic.

The AUCs, 95% CIs and asymptotic significance of receiver operating characteristic curves for EMT, endometrial volume and UP on clinical pregnancy can be observed from Table 3 and Figure 3. The AUC values of EMT and endometrial volume on the endometrial transformation day were 0.461 and 0.458, while those 1 day before ET were 0.501 and 0.444, respectively, revealing that neither EMT nor endometrial volume could well predict the clinical pregnancy outcomes in ADM patients undergoing FET. However, the AUC values of UP were 0.720 and 0.627 on the 2 time points.

**Table 3:** Receiver operating characteristic curves of EMT, endometrial volume, and uterine peristalsis on clinical pregnancy of ADM.

Value	Area	Asymptotic significance	95% CI
Endometrial thickness on the day of endometrial transformation	0.461	0.404	0.372–0.550
Endometrial volume on the day of endometrial transformation	0.458	0.362	0.366–0.549
Endometrial thickness on the day before embryo transfer	0.501	0.987	0.410–0.591
Endometrial volume on the day before embryo transfer	0.444	0.225	0.354–0.533
Uterine peristalsis on the day of endometrial transformation	0.720	0.001	0.643–0.797
Uterine peristalsis on the day before embryo transfer	0.627	0.006	0.541–0.712

ADM = adenomyosis, CI = confidence interval, EMT = endometrial thickness.

## 4. Discussion

Effective assessment of endometrial receptivity and improvement of the predictive efficacy for pregnancy outcomes in patients can help enhance the patient life quality and reduce the burden of adverse pregnancy outcomes on them. Ultrasound technology is a fast, economical and simple method for evaluating endometrial receptivity. EMT is the most common endometrial receptivity parameter measured by transvaginal ultrasound, which can represent the endometrial growth status. Some scholars believe that the remarkably elevated pregnancy rates in the case of higher EMT are not related to the quantity and quality of embryos transferred.^[9,21]^ However, some authors suggest that EMT is not related to pregnancy rate.^[22]^ No convincing conclusion can be drawn on the relation of EMT with pregnancy rate in IVF, and EMT cannot be used as a basis for deciding to cancel the cycle or perform the freeze-all strategy.^[23]^

As discovered by Bahar et al, EMT of women with live birth and women with no live birth was similar after fresh ET or FET. Even without a critical value, EMT was not linearly correlated with the live birth or miscarriage rates. Therefore, they concluded that after excluding the thinner EMT and intrauterine lesions, ET should not be refused.^[24]^ This research investigated the prediction value of EMT in FET for the pregnancy outcome in patients with ADM. EMT was not significantly different between the 2 groups on the endometrial transformation day and 1 day before ET, suggesting that EMT was not the key factor influencing pregnancy outcomes. Furthermore, relative to an EMT > 8 mm, the pregnancy rate of FET patients with an EMT of 7 to 8 mm 1 day before ET did not significantly decrease.

The EMT determined through transvaginal 2D ultrasound does not reflect the endometrial volume. In comparison, the endometrial volume determined by 3D ultrasound exhibits the higher accuracy and repeatability.^[25]^ Three-dimensional ultrasound can be used as a tool for ART and for predicting the success rate of pregnancy.^[26]^ It has been reported that the acceptable volume of endometrial receptivity is 3.2 mL, and the negative predictive value of the freezing–thawing cycle for establishing pregnancy is 96%, while the positive predictive value is only 28.6%.^[27]^ In some studies, endometrial volume instead of EMT is utilized to predict endometrial receptivity. However, whether endometrial volume can be used to predict pregnancy remains controversial. No matter whether the measurement is conducted on the oocyte retrieval day or human chorionic gonadotropic beta measurement day, endometrial volume does not predict the pregnancy outcome. It is probably because that, apart from indicating the endometrial volume, it is mostly influenced by uterine volume.^[28,29]^ Our main finding indicated no significant difference in the anterior–posterior diameter of the uterus in ADM patients between the pregnant and nonpregnant groups. Moreover, endometrial volume on the progesterone administration day and 1 day prior to ET was not significantly different. Assen et al examined 142 women and suggested that the endometrial volume evaluated through 3D transvaginal ultrasound could not well predict pregnancy during the ET cycle of a single blastocyst, with the AUC of 0.48.^[30]^ This is consistent with our research results.

For ADM patients, infertility is probably related to local endometrial inflammation, in particular in the case of lesions penetrating inner myometrium. After the ADM focus is formed, platelets will aggregate and hypoxia is induced, thus stimulating the generation of inflammatory factors, prostaglandins, and local estrogen.^[13,31]^ Finally, uterine hyperperistalsis will be caused. Abnormal contractions have been suggested to destroy gamete and embryo transport, thus affecting the pregnancy rate of ADM patients, but such fibrosis seems not to cause any changes in the EMT or endometrial volume.

In the periovulatory phase, endometrial waves are usually retrograde, thereby allowing active sperm transport from the cervix to fallopian tubes, whereas opposite waves occur following ovulation, which may avoid embryo expelling from the cervix or tubes while allowing the optimal conception product placement prior to implantation. At last, the peristaltic activity nearly disappears in the secretory phase, thus not blocking implantation.^[32]^ Therefore, in the binary logistic regression, peristaltic wave 1 day before ET was not the independent influencing factor for pregnancy outcome, and the AUC was smaller than the predictability of endometrial peristaltic wave on the endometrial transformation day for the pregnancy outcome.

Some limitations should be noted in the present work. For example, due to the limited sample size, this study did not explore the influences of EMT < 7 mm on clinical pregnancy and the impacts of EMT and endometrial volume on live birth rate among ADM cases receiving FET. Consequently, further large prospective studies should be conducted to assess the influences of EMT and endometrial volume on live birth rate following frozen transfer of a single blastocyst.

## 5. Conclusion

Collectively, when measured on the progesterone administration day and 1 day before ET, neither the EMT nor endometrial volume is significantly different between the pregnant and nonpregnant groups. Further, uterine hyperperistalsis can be used to forecast unfavorable pregnancy outcomes in ADM patients.

## Acknowledgments

The authors thank the staffs of the Department of Chengdu Women’s and Children’s Central Hospital for their cooperation and support.

## Author contributions

**Data curation:** Xuefei Liang.

**Formal analysis:** Meixian Wang.

**Investigation:** Xue Ke.

**Writing – original draft:** Xue Ke.

**Writing – review & editing:** Xiaoguang Shao.
